# Widespread Traces of Lytic Kaposi Sarcoma-Associated Herpesvirus in Primary Effusion Lymphoma at Single-Cell Resolution

**DOI:** 10.1128/MRA.00851-20

**Published:** 2020-11-05

**Authors:** Nicole C. Rondeau, Michael O. Finlayson, JJ L. Miranda

**Affiliations:** a Department of Biology, Barnard College, Columbia University, New York, New York, USA; b JP Sulzberger Columbia Genome Center, Columbia University Irving Medical Center, New York, New York, USA; Indiana University, Bloomington

## Abstract

Cancer cells of primary effusion lymphoma (PEL) often contain both Kaposi sarcoma-associated herpesvirus (KSHV) and Epstein-Barr virus (EBV). We measured the interplay of human, KSHV, and EBV transcription in a cell culture model of PEL using single-cell RNA sequencing. The data detect widespread trace expression of lytic KSHV genes.

## ANNOUNCEMENT

Immortalized cell lines derived from primary effusion lymphoma (PEL) positive for both Kaposi sarcoma-associated herpesvirus (KSHV) and Epstein-Barr virus (EBV) serve as tractable systems for studying transcriptional host-pathogen interactions. We grew BC-1 cells (1) in culture (2) and processed log-phase samples for single-cell RNA deep sequencing (scRNA-seq) with Gel beads in EMulsion microfluidic (GEM) technology (3) using a Chromium Single Cell 3′ Library & Gel Bead Kit v2 (10X Genomics, Pleasanton, CA). The experiment was repeated twice with two independent biological replicates that each yielded ∼400 to 700 million paired-end reads of 28 to 101 nucleotides.

We built a bioinformatics pipeline to simultaneously measure human and viral transcription. Reads were demultiplexed and aligned using Cell Ranger (10X Genomics). A custom genome reference was created by appending the viral genomes of EBV (4) (GenBank accession number NC_007605.1) and KSHV (5) (GenBank accession number U75698.1) to the human genome GRCh38. Reads aligning to viral genomes were assigned to features using a heuristic algorithm implemented in Python (https://github.com/jjmirandalab/scrnaseq). Feature definitions were obtained from a published EBV annotation (6) (https://github.com/flemingtonlab/public/blob/master/annotation/chrEBV_B95_8_Raji.ann) and downloaded from the KSHV reference (5) (GenBank accession number U75698.1). First, each virus-mapping read was assigned the union of all RNA genome features its alignment overlapped with. Second, each molecule was assigned the intersection of all feature assignments from reads with its corresponding unique molecular identifier (UMI). If any molecule mapped to multiple genomes, it was excluded. Finally, for each unique set of features assigned to a molecule, counts were recorded for each cell. Cells were filtered using R scripts (https://github.com/jjmirandalab/scrnaseq). Distributions of cell quality metrics were modeled as log-normal for the total UMI count, log-normal for the proportion of expression from mitochondrial genes, and normal for the number of genes detected. Cells with extreme metric values were then identified according to the fit distributions. Data between samples were normalized with sctransform (7). “Sample” was included as the “batch_var” parameter.

Earlier RNA-seq methods only measured expression of exon regions that did not overlap another transcript (8, 9); our approach discards less data. Approximately 20% of the feature sets we count correspond to potentially overlapping transcripts that would have previously been ignored. Comprehensive profiling instills more confidence in our interpretations.

Preliminary analysis of our scRNA-seq data reveals an unexpected plethora of lytic KSHV transcripts. Herpesviruses switch between a transcriptionally quiescent latent state and a reactivated lytic state. Genes were classified as latent or lytic based on expression timing (10, 11). For EBV, we detected only latent genes. For KSHV, we detected both latent and lytic genes (Table 1). Transcription of the lytic Open Reading Frames 16, K9, K4, 54, 45, and 75, as well as the lytic polyadenylated nuclear RNA PAN gene, can each be found in ∼5 to 50% of cells. While we usually detected more than 1 count in each cell for the latent genes LANA and vIL6, lytic genes appear with only 1 count most of the time. Greater than 50% of cells contain these trace levels of lytic RNA. In contrast, ∼98% of BC-1 cells are thought to harbor latent KSHV because lytic protein is not detected (12). Although more detailed analysis is required, our observations prompt a thoughtful redefinition of a “latent” transcriptome.

**TABLE 1:** Detection of KSHV and EBV transcripts in BC-1 PEL cells with scRNA-seq

Transcript	Positive cells (% of total)^*a*^		Cells with 1 count (% of positive)	
	Replicate 1	Replicate 2	Replicate 1	Replicate 2
EBV latent				
A73, BARF0, RPMS1^*b*^	29.8	30.4	69.7	71.7
EBER-2	4.4	6.6	95.3	97.4
EBNA-1 Q_p_	1.1	0.4	97.0	100.0
KSHV latent				
ORF K2 vIL6	98.7	91.0	2.9	14.8
ORF 72 vCyclin	2.1	1.9	98.4	94.6
ORF 73 LANA	99.9	99.7	0.2	0.4
KSHV lytic				
ORF 11	1.1	0.3	80.6	70.0
ORF K3	1.1	0.8	83.9	87.0
ORF K4	16.4	9.9	76.7	85.5
ORF K5	5.2	2.7	83.3	87.2
ORF K6	3.3	0.8	87.2	82.6
ORF 16	45.6	31.2	70.4	80.5
ORF 45	10.4	5.0	87.7	94.5
ORF 47	1.5	0.2	88.1	100.0
ORF 54	11.0	6.8	71.6	75.5
ORF K9	38.2	19.7	63.1	81.1
ORF K10	5.8	2.8	90.4	97.6
ORF K11	1.2	0.3	88.2	100.0
ORF 75	9.7	5.7	90.0	93.5
PAN	16.3	6.8	87.2	92.9
KSHV unclassified				
ORF K2 vIL6, ORF 2^*c*^	2.3	1.1	95.5	87.5
ORF 2^*d*^	6.2	2.4	90.4	98.6

a Only transcripts present in at least 1% of cells in at least one replicate are shown.

b The union of transcripts could be assigned to multiple genes, but all components are latent.

c The union of transcripts contains one component of unknown expression timing.

d The expression timing of this transcript is not known.

### Data availability.

Our data have been deposited in the NCBI Gene Expression Omnibus (13, 14) under accession number GSE154900.
